# Utilizing Biotinylated Proteins Expressed in Yeast to Visualize DNA–Protein Interactions at the Single-Molecule Level

**DOI:** 10.3389/fmicb.2017.02062

**Published:** 2017-10-24

**Authors:** Huijun Xue, Yuanyuan Bei, Zhengyan Zhan, Xiuqiang Chen, Xin Xu, Yu V. Fu

**Affiliations:** ^1^State Key Laboratory of Microbial Resources, Institute of Microbiology, Chinese Academy of Sciences, Beijing, China; ^2^Savaid Medical School, University of Chinese Academy of Sciences, Beijing, China

**Keywords:** biotin-streptavidin, BirA-Avi, budding yeast, DNA-protein interaction, quantum dots, total internal reflection fluorescence microscopy

## Abstract

Much of our knowledge in conventional biochemistry has derived from bulk assays. However, many stochastic processes and transient intermediates are hidden when averaged over the ensemble. The powerful technique of single-molecule fluorescence microscopy has made great contributions to the understanding of life processes that are inaccessible when using traditional approaches. In single-molecule studies, quantum dots (Qdots) have several unique advantages over other fluorescent probes, such as high brightness, extremely high photostability, and large Stokes shift, thus allowing long-time observation and improved signal-to-noise ratios. So far, however, there is no convenient way to label proteins purified from budding yeast with Qdots. Based on BirA–Avi and biotin–streptavidin systems, we have established a simple method to acquire a Qdot-labeled protein and visualize its interaction with DNA using total internal reflection fluorescence microscopy. For proof-of-concept, we chose replication protein A (RPA) and origin recognition complex (ORC) as the proteins of interest. Proteins were purified from budding yeast with high biotinylation efficiency and rapidly labeled with streptavidin-coated Qdots. Interactions between proteins and DNA were observed successfully at the single-molecule level.

## Introduction

Understanding the dynamic complexity of life process is one of the major goals in molecular biology. Interactions between nucleic acids and proteins are essential in many important biochemical reactions.

In recent years, the rapidly developing technique of single-molecule fluorescence microscopy has made great contributions to revealing the details of nucleic acid and protein interactions in various biological processes, such as DNA replication (Yardimci et al., 2012; Duzdevich et al., 2015; Ticau et al., 2015; Graham et al., 2017), spliceosome (Fareh et al., 2016), and CRISPR-Cas systems (Redding et al., 2015).

In single-molecule studies, most biomolecules should be labeled with proper fluorescent probes. Accordingly, the development of fluorescent probes and labeling techniques plays a key role in single-molecule fluorescence detection. In general, there are three classes of fluorescent probes: (1) organic dyes; (2) fluorescent proteins; and (3) quantum dots (Qdots) (Stratmann and van Oijen, 2014). Qdots are tiny light-emitting semiconductor particles of nanometer scale and have emerged as a new class of fluorescent probe (Alivisatos et al., 2005; Xing et al., 2007). Compared with organic dyes and fluorescent proteins, Qdots have several unique merits, including intense brightness, exceptional photostability, and large Stokes shift. Thus, they are suitable for long-time observation and enable signal-to-noise ratio improvement (Medintz et al., 2005; Nelson et al., 2011).

To label a specific protein with Qdots, one strategy involves conjugating Qdots with the corresponding antibody. Several antibody labeling methods have been developed, including covalent crosslinking and noncovalent biotin–avidin binding (Xing et al., 2007). *N*-(3-dimethylaminopropyl)-*N*′-ethylcarbodiimide) (EDC) hydrochloride conjugation is one of the covalent crosslinking methods used to achieve Qdot-antibodies by conjugating carboxylic-acid-coated Qdots and the primary amines of antibodies in the presence of catalyst EDC. However, large aggregations of crosslinking antibodies and Qdots are often produced by this method. In another method, antibodies are labeled by conjugating carboxylic-acid-coated Qdots with free and accessible sulfhydryl groups. Even though this method is better than EDC hydrochloride conjugation for antibody labeling, it is not applicable for all proteins because some proteins do not have a sulfhydryl group. The site-specific covalent crosslinking method of acquiring Qdot-antibodies involves conjugating Qdots and antibodies via reactive aldehyde groups through oxidation of carbohydrate groups on Fc portions of the antibodies. However, this site-specific method is only suitable for antibody labeling because it is rare for proteins having site-specific carbohydrate groups. The biotin–avidin binding method involves conjugating biotinylated antibodies and streptavidin-coated Qdots; this method relies on the strong and rapid noncovalent biotin–streptavidin interaction (Kd of 4 × 10^−14^ M) (Green, 1990). This method is not only used for antibody labeling but has also been proved to be an elegant method to label target proteins with Qdots (Redding et al., 2015).

The biotin-labeling protein techniques can be divided into two classes: (1) chemical crosslinking and (2) genetic editing. Both the primary amino and the sulfhydryl group labeling methods provide two widespread and convenient chemical crosslinking ways to biotinylate a protein of interest. However, chemical crosslinking is not site-specific, and this limits its utilization in most experiments. A method for site-specific biotinylated protein can be achieved via labeling the target protein with a Sort tag by genetic editing. The labeling process is complex and includes three essential steps: (1) purification of the Sort-tagged target protein; (2) biotinylation of the target protein via incubation with the Sortase and biotinylated peptide (biotin-LPETGG); and (3) purification of the biotinylated-Sort-tagged target protein to remove Sortase and excessive biotinylated peptides (Duzdevich et al., 2015). An alternative site-specific biotinylation method is based on the BirA–Avi system, in which biotin ligase BirA from *Escherichia coli* is used to catalyze Avi-tag to be biotinylated (Beckett et al., 1999). A combination of the BirA–Avi and biotin–streptavidin systems has been used to purify protein from *Arabidopsis thaliana, Drosophila* embryos, and mammalian cells owing to site-specific biotinylation and a strong biotin–streptavidin noncovalent interaction (de Boer et al., 2003; Deal and Henikoff, 2011; Strubbe et al., 2011). In budding yeast, this combination system was used to improve the sensitivity of chromatin immunoprecipitation (van Werven and Timmers, 2006); however, to our knowledge, no study of biotinylated protein purification has been reported so far.

DNA replication is a dynamic and complex process, involving a large number of proteins. Based on biochemistry and molecular biology and with the aid of structural techniques, the process of DNA replication and its key proteins have been elucidated over the past decade (Sun et al., 2013, 2014; Yeeles et al., 2015; Bell and Labib, 2016; Burgers and Kunkel, 2017; Coster and Diffley, 2017; Zhou et al., 2017). However, many details such as the loading and unwinding mechanisms of eukaryotic DNA helicase CMG (Cdc45-MCM-GINS) and the action of leading- and lagging-strand DNA polymerases in the replisome, are still obscure. Powerful single-molecule techniques, especially total internal reflection fluorescence microscopy (TIRFM), have made it possible to visualize this complex process based on living cells, cell-free extract systems, and purified protein systems (Yao et al., 2009; Reyes-Lamothe et al., 2010; Fu et al., 2011; Duzdevich et al., 2015; Ticau et al., 2015, 2017; Graham et al., 2017). Research in budding yeast indicates that the use of a purified protein system would be more beneficial in studying the characteristics of the target proteins in budding yeast, owing to yeast auto-fluorescence and low labeling efficiency of target proteins in yeast extracts (Billinton and Knight, 2001; Duzdevich et al., 2015). Several exciting insights into DNA replication in budding yeast have recently been achieved using organic dyes and Qdot-labeled purified proteins (Duzdevich et al., 2015; Ticau et al., 2015, 2017). Organic dyes are beneficial for quantitative analysis, and Qdots allow long-term observation (from minutes to hours). However, to date, there is no convenient way to label proteins purified from budding yeast with Qdots.

In this study, we present a method to achieve a Qdot-labeled target protein and image it by TIRFM at the single-molecule level. Two budding yeast strains bearing *BIRA* or *NLS-BIRA* in the genome and two universal expressing plasmids with Avi-tags at the N- or C-termini were constructed. As proof-of-concept, we chose the replication protein A (RPA), the single-strand binding protein, and the origin recognition complex (ORC), a site-specific double-strand binding protein. ORC and RPA showing high levels of biotinylation were purified from budding yeast, and the DNA–protein interactions were successfully observed using TIRFM with the aid of streptavidin-coated-Qdots.

## Materials and methods

### Plasmid construction

To express the BirA enzyme in budding yeast, YIpLac128-*GAL1-BIRA* and YIpLac128-*GAL1-NLS-BIRA* were constructed: (1) *BIRA* was amplified from pBirAcm extracted from strain AVB_101_ (AVB 101, Avidity), and *NLS-BIRA* was amplified using a primer containing a nuclear localization signal (NLS) sequence from the SV40 large T-antigen (van Werven and Timmers, 2006); (2) *BIRA* and *NLS-BIRA* were then inserted into pYES2.0 containing a *GAL1* promoter between the *Hind*III and *Bam*HI sites using a quick-fusion cloning kit (Biotool); and (3) finally, *GAL1-BIRA/GAL1-NLS-BIRA* was amplified and cloned between *Hind*III and *Bam*HI sites in YIpLac128.

pRS306/*RFA2-Gal-RFA3* and pRS303/*GAL4*-*CBP-TEV-RFA1* are used for RPA expression (Yeeles et al., 2015), and pJF17, pJF18, and pJF19 are used for ORC expression (Frigola et al., 2013). To label RPA and ORC with Avi-tag, *3*×*FLAG-AVI* and *AVI* were added at the C-terminus of *RFA2* in the plasmid pRS306/*RFA2-Gal-RFA3* and at the N-terminus of *ORC1* before *CBP* in the plasmid pJF19 using overlap polymerase chain reaction (PCR) as described by Wang et al. (2017) and subcloned; the two new plasmids were named pC-AVI and pN-AVI (Figures S1, S2). pC-AVI was constructed with several detailed steps: (1) *3*×*FLAG-AVI* fragment was amplified from pFA6a-*3*×*FLAG-AVI* using FLAG-AVI-F/R primers, and *RFA2* gene and a terminator were amplified from pRS306/*RFA2-Gal-RFA3* using RFA2-F/R and terminator-F/R primers, respectively; (2) *RFA2-3*×*FLAG-AVI* was amplified by overlap PCR with RFA2-F/FLAG-AVI-R primers; (3) *RFA2-3*×*FLAG-AVI-*terminator was amplified by overlap PCR with Final-F/R primers (Figure S1A); and (4) the final fragment was inserted into *Asc*I-*Xho*I digested pRS306/*RFA2-Gal-RFA3* by homologous recombination using a quick-fusion cloning kit (B22611, Biotool). For pN-AVI construction several steps were followed: (1) *AVI-CBP* was first amplified from pJF19 by overlap PCR with AVI-CBP-F1/AVI-CBP-R and AVI-CBP-F2/AVI-CBP-R primers; (2) *CBP-ORC1*-terminator was amplified from pJF19 using ORC1-terminator-F/R primers; (3) *AVI*-*CBP-ORC1*-terminator was amplified by overlap PCR with Final-F/R primers (Figure S2A); and (4) the final fragment was inserted into *Asc*I-*Xho*I digested pJF19 by homologous recombination (B22611, Biotool). The related primers can be seen in Table S1.

### Yeast strains

yRH100 is an isogenic derivative of W303-10D strain (Heller et al., 2011). A strain for protein expression was constructed by *pep4* knock-out in yRH100, and named yFYV3 (Table 1).

**Table 1:** Yeast strains used in this study.

**Strains**	**Genotype**
yRH100	*MATa ade2-1 ura3-1 his3-11,15 trp1-1 leu2-3,112* *can1-100 lys2::hisG bar1::hisG*
yFYV3	same as yRH100, but *pep4::NATMX6*
yFYV4	same as yFYV3, but *leu::LEU2YIplac128/GAL1-BIRA*
yFYV5	same as yFYV3, but *leu::LEU2YIplac128/GAL1-NLS-BIRA*
yFYV6	same as yFYV3, but *his3::HIS3pRS303/CBP-TEV-RFA1, GAL4* *ura3::URA3pC-AVI/RFA3, RFA2-FLAGAVI*
yFYV7	same as yFYV4, but *his3::HIS3pRS303/CBP-TEV-RFA1, GAL4* *ura3::URA3pC-AVI/RFA3, RFA2-FLAGAVI*
yFYV8	same as yFYV5, but *his3-11::HIS3pJF17/ORC3,ORC4* *trp1-1::TRP1pJF18/ORC5,ORC6 ura3-1::URA3pN-AVI/AVI-CBP-ORC1,ORC2*

### Protein expression and purification

Both RPA and ORC were induced as described in several publications (Frigola et al., 2013; Yeeles et al., 2015), except that cells were cultured in YP-glycerol instead of YP-raffinose (Heller et al., 2011).

ORC was purified using calmodulin affinity resin (17-0529-01, GE Healthcare) by affinity purification following the protocol described by Frigola et al. (2013). RPA purification was based on the protocol by Yeeles et al. (2015), except that the heparin column and MonoQ column were replaced with ANTI-FLAG® M2 affinity gel (A2220-5ML, Sigma) and a Superdex 200 10/300 GL column (17-5175-01, GE Healthcare), respectively. Cell powder was thawed in a water bath and resuspended thoroughly using binding buffer (25 mM Tris-HCl pH 7.2, 10% glycerol, 1 mM DTT, 500 mM NaCl) with protease inhibitor cocktail at 1:100 volumn:volumn ratio (DI101-02, TransGen, China). The whole-cell extract was separated with insoluble materials by ultra-centrifugation (235,000 g, 4°C, 1 h), incubated, and rotated with 1.5 ml calmodulin affinity resin (786-282, G-bioscience) together with 2 mM CaCl_2_ at 4°C for approximately 90 min. Resin was collected using a 10 ml empty column (29925, Thermo Fisher Scientific) and washed extensively with binding buffer containing 2 mM CaCl_2_. Bound proteins were eluted using binding buffer supplement with 2 mM EDTA and 2 mM EGTA. Peak fractions were pooled and incubated with 1.5 ml anti-Flag M2 affinity gel pre-equilibrated using binding buffer for 90 min at 4°C with rotation. Resin was collected with a 10 ml empty column and also washed extensively with binding buffer. Bound proteins were eluted in 3 CV binding buffer, with 0.5 mg/ml 3 × Flag peptide. The peak fractions were pooled, concentrated, and injected onto a Superdex 200 10/300 GL column. The fraction containing RPA was pooled and mixed with binding buffer containing 66% glycerol and 1 mM EDTA at 1:1 volume ratio, and aliquoted and then stored at −80°C.

### Biotinylation detection and efficiency evaluation

The biotinylation of target proteins was detected using streptavidin-horseradish peroxidase (HRP); the extent of biotinylation was evaluated using a streptavidin gel shift assay as described by van Werven and Timmers (2006). Boiled whole-protein extracts or purified protein samples were incubated with unboiled streptavidin for 5 min at room temperature and then loaded onto a sodium dodecyl sulfate-polyacrylamide gel. For whole-protein extracts, the gel was transferred to a polyvinylidene fluoride membrane and analyzed using immunoblotting. The antibodies used in this study were CBP tag antibody (A01798, Genscript), Flag tag antibody (F3165, Sigma-Aldrich), and streptavidin poly-HRP (21140, Thermo Fisher Scientific). For purified proteins, the gel was stained using Coomassie Brilliant Blue, and the intensity of the bands was quantified using Image J.

### Biotinylated ssDNA preparation for single-molecule assay

Biotinylated single-strand DNA (ssDNA) was prepared by rolling circle DNA replication (RCR) as described in several publications (Gibb et al., 2012; Qi and Greene, 2016), except that phi 29 DNA polymerase was bought from Thermo Fisher Scientific (EP0091), RCR reaction time was extended to 18 h, and the biotinylated primer was synthesized by Sangon Biotech, China.

### λ-ARS1/λ-ARS1-ARS609 DNA preparation and biotinylation

To obtain λ-ARS1 DNA, an 838 bp DNA fragment containing an autonomously replicating sequence (ARS) 1 sequence was amplified from budding yeast genomic DNA of yRH100 and integrated into 48,502 bp native lambda DNA (NEB) at the *Xba*I site using homologous recombination (B22611, Biotool). The recombination product was then packaged using MaxPlax™ Lambda Packaging Extracts (MP5110, Epicentre), and the positive plaque was confirmed by PCR and sequencing. Finally, λ-ARS1 DNA (49,340 bp in length) was prepared from liquid lysates. λ-ARS1-ARS609 DNA, which was 50,055 bp in length, was obtained using the same strategy by integrating a 715 bp DNA fragment containing ARS609 sequence in the *Xho*I site of λ-ARS1. λ-ARS1/λ-ARS1-ARS609 were biotinylated following the protocol by Yardimci et al. (2012), and the biotinylated primers were synthesized by Invitrogen (Thermo Fisher Scientific).

### Single-molecule imaging and data analysis

Single-molecule imaging was performed on an Olympus IX-71 inverted TIRFM microscope using a 60× oil objective (numerical aperture = 1.49). A 405 nm or 532 nm laser was used for fluorescent probe excitation and W-View Gemini Imaging splitting optics (Hamamatsu photonics K.K.) were used to produce dual wavelength images. Image sequences were recorded using an electron-multiplying charge-coupled device (EM-CCD) (Andor). DNA–protein interactions were performed in flow cells as in previous studies (Fu et al., 2011; Yardimci et al., 2012; Chen et al., 2017). Briefly, the coverslip was cleaned, silane-cured, and functionalized using partially biotinylated polyethylene glycol (PEG); a glass slide with two holes was secured to a functionalized coverslip using a piece of double-sided tape with a rectangular channel in the center to construct a chamber. The inlet and outlet tubing pieces were inserted into the holes for buffer-in and buffer-out. Biotinylated DNA was injected with blocking buffer (20 mM Tris pH 7.5, 50 mM NaCl, 2 mM EDTA, 0.2 mg/ml BSA) at a rate of 50 μl/min. Proteins were labeled using streptavidin-coated Qdot_705_ (Q10163MP, Thermo Fisher Scientific), which was excited using a 405 nm laser. RPA was injected with blocking buffer, and ORC was injected with binding buffer (25 mM HEPES pH 7.6, 12 mM MgOAc, 50 μM ZnOAc, 1 mM DTT, 225 mM KGlut, 3 mM ATP, 0.2 mg/ml BSA) at the rate of 10 μl/min. Double-strand DNA was stained using SYTOX Orange (S11368, Thermo Fisher Scientific), which was excited using a 532 nm laser.

The imaging sequences were obtained with 100 ms exposure time, 100 ms interval time, 200 electron-multiplying gain, and 100 μl/min flow. Images were analyzed using Fiji (Schindelin et al., 2012). For the images of RPA binding on single-strand DNA, 51 sequential images from its corresponding stack were processed by the average intensity Z projection type. For the ORC binding on dsDNA images, 61 sequential images from its corresponding stack were processed by average intensity type of Z projection. The distributions of ORC binding on ARS inserted λDNA were measured manually, and data were analyzed using R and GraphPad Prism; an error was defined on the basis of 1,000 bootstrap samples and a 95% confidential interval.

## Results

### Construction of protein biotinylation system

**Figure 1:**
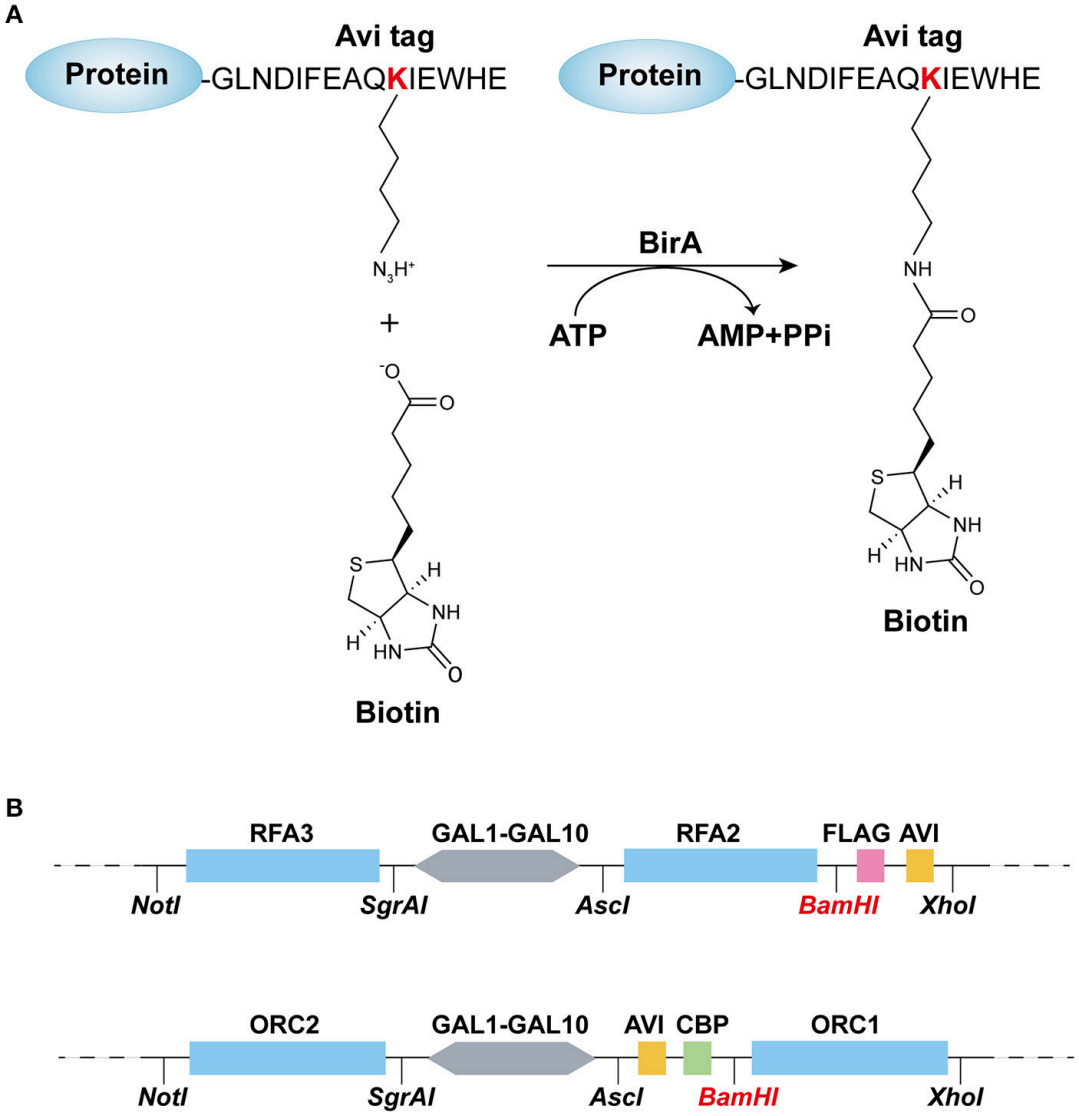
Recombination-protein biotinylated system established in budding yeast. **(A)** Avi-tagged target protein biotinylated by BirA. **(B)** Main elements and enzyme sites of (top) pC-AVI and (bottom) pN-AVI. The two plasmid maps are given in Figures S1, S2.

The biotin carboxyl carrier protein (BCCP) subunit of acetyl-CoA carboxylase can be biotinylated by the biotin holoenzyme synthetase, BirA, at the epsilon amino group of a specific lysine residue in *E. coli*. Based on this fact, the minimal 15-mer GLNDIFEAQKIEWHE Avi-tag was designed for BirA-catalyzed biotinylation (Beckett et al., 1999) as shown in Figure 1A. To acquire the biotinylated proteins in budding yeast, in this study, we combined the BirA–Avi and *GAL1-GAL10* promoter-driven expression systems to establish a convenient method for obtaining biotinylated target proteins in budding yeast. To produce the biotinylated target proteins *in vivo*, YIpLac128-*GAL1-BIRA* and YIpLac128-*GAL1-NLS-BIRA* were integrated into yFYV3 to obtain the new strains (yFYV4 and yFYV5, respectively; Table 1). For the proof-of-concept, we chose RPA and ORC as the proteins of interest.

RPA, a single-strand binding protein, is ubiquitous and conserved in eukaryotes. It is known to be involved in many essential DNA metabolic pathways such as DNA replication and homolog recombination (Fanning et al., 2006). In budding yeast, RPA is encoded by three essential genes, *RFA1, RFA2*, and *RFA3*. To obtain biotinylated RPA, the plasmid pRS306/*RFA2-GAL-RFA3* (Yeeles et al., 2015) was reconstructed by replacing *RFA2* with *RFA2-3*×*FLAG-AVI* and named pC-AVI (Figure 1B, top panel, Figure S1B). Both pRS303/*GAL4-CBP-TEV-RFA1* and pC-AVI were integrated into yFYV4 to obtain yFYV7. The two plasmids were also integrated into yFYV3 to obtain the negative control strain, yFYV6 (Table 1).

ORC, the origin recognition complex, can recognize and bind to replication originating DNA, autonomously replicating sequences (ARS) in the presence of ATP, both *in vivo* and *in vitro* (Bell and Labib, 2016). ORC is encoded by six essential genes in budding yeast, including *ORC1–6* in budding yeast. *CBP-ORC1* in pJF19 (Frigola et al., 2013) was replaced with *AVICBP-ORC1* to produce a new plasmid, named pN-AVI (Figure 1B, lower panel, Figure S2B). Since ORC recognizes and binds to nuclear origin DNA in the G1 phase, a strain for purifying biotinylated ORC was constructed by integrating pJF17, pJF18, and pN-AVI into strain yFYV5 bearing *NLS-BIRA*, and then named yFYV8. Accordingly, using α factor, yeast cells can be easily arrested at G1 phase due to the *bar1* mutation, and a high extent of ORC biotinylation can be obtained with nuclear NLS-BirA (Table 1).

In both pN-AVI and pC-AVI plasmids, a *Bam*HI enzyme site was introduced between the gene of interest and the *AVICBP* or *FLAGAVI* fragment; thus, it is easy to replace the existing gene with another gene of interest. Taken together, a budding yeast biotinylated system, including two strains integrated in the genome with *BIRA* or *NLS-BIRA* and two universal plasmids with N-terminal Avi-tag or C-terminal Avi-tag, were constructed.

### Biotinylation efficiency of Avi-Tagged proteins

To validate the feasibility of our biotinylated system, we examined Rfa2 and Orc1 biotinylation *in vivo* and evaluated the biotinylation efficiency by immunoblotting analyses. Yeast cells were grown to mid-log phase, then *GAL1,10* driving expression was induced by adding 2% galactose. Whole-protein extracts of yFYV3 and yFYV6–8 were prepared in order to analyze Avi-tagged protein biotinylation. Biotinylation of the proteins were detected using streptavidin-HRP, which can specifically recognize the biotin molecule ligated on the Avi-tag. Biotinylation efficiency was analyzed using anti-Flag antibody for Rfa2 and anti-CBP antibody for Orc1.

**Figure 2:**
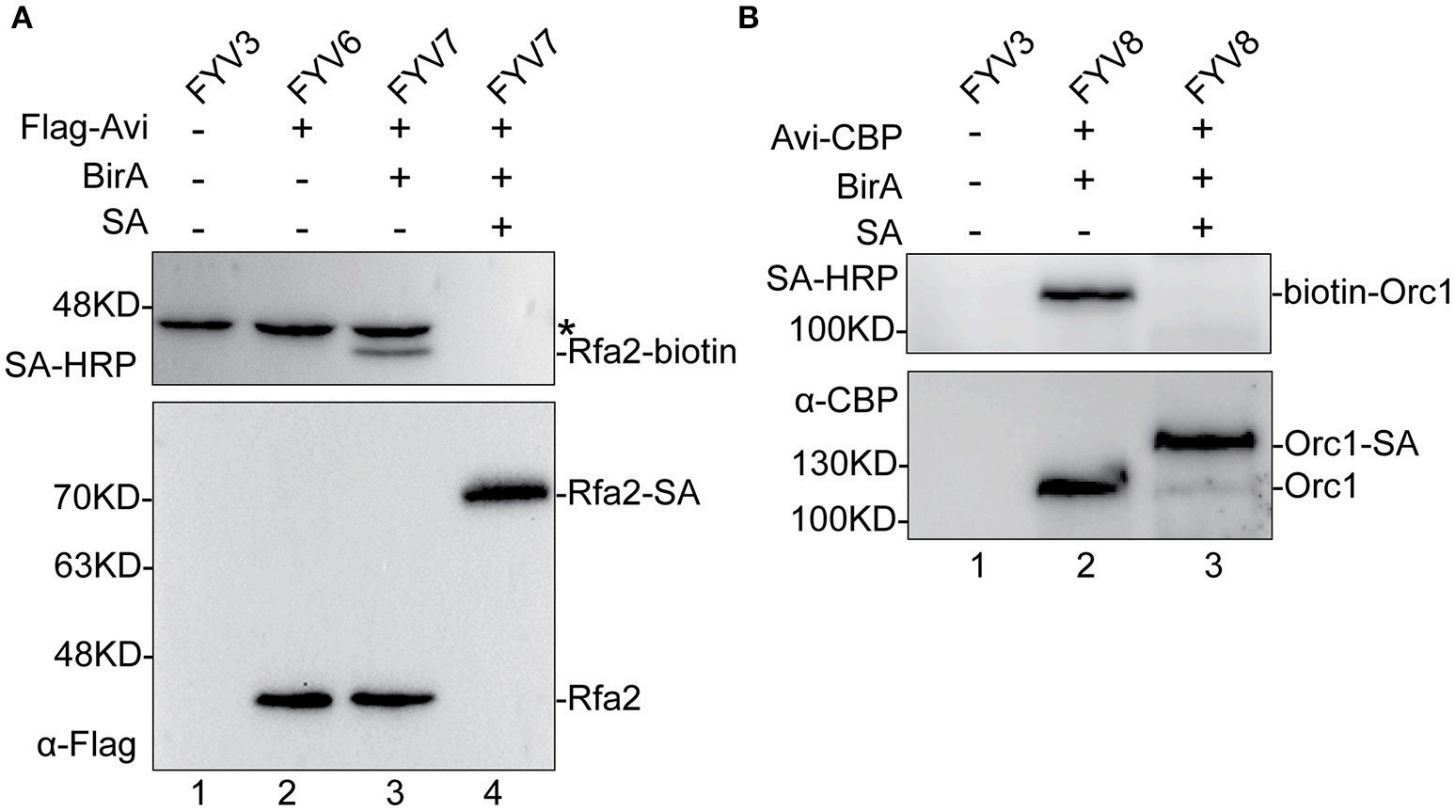
Detection and evaluation of biotinylation of Rfa2 and Orc1 using Western blotting. Whole-protein extracts of yFYV3, yFYV6, yFYV7, and yFYV8 were used to analyze the biotinylation of target proteins. **(A)** Rfa2 biotinylation by BirA *in vivo* was successfully detected using streptavidin-HRP in lane 3 (top). The bands in lanes 1–3 (top), which are most likely Arc1, are indicated by an asterisk. Biotinylated Rfa2 (Rfa2-biotin) was almost totally shifted in the presence of streptavidin in lane 4, which was analyzed using streptavidin-HRP (top) and anti-Flag antibody (bottom). **(B)** Orc1 biotinylation. Biotinylated Orc1 (biotin-Orc1) was detected successfully in lane 2 (top), and 91% Orc1 was shifted in the presence of streptavidin (SA) in lane 3 (bottom), which was quantified using Image J.

In Figure 2A, as can be seen by comparing lane 3 with lane 2, biotinylated Rfa2 was successfully detected by streptavidin-HRP in lane 3, suggesting that Avi-tagged Rfa2 was biotinylated by BirA *in vivo*. Except for Rfa2, another band in lane 1–3 were detected by streptavidin-HRP either in the absence of BirA (lane 2) or in the absence of both BirA and Avi tag (lane 1). This protein is most likely Arc1, which is a 45 kDa endogenous biotinylated protein in budding yeast (Kim et al., 2004). Biotinylation of Rfa2 was further confirmed by the streptavidin gel shift assay (Figure 2A, lanes 3 and 4). If Rfa2 is biotinylated, incubation with streptavidin will cause a shift of the biotinylated Rfa2 on the gel. In lane 4 of Figure 2A, immunoblotting with both streptavidin-HRP and anti-Flag antibody showed that biotinylated Rfa2 was totally up-shifted in the presence of streptavidin; these data indicate that the biotinylation efficiency of RPA was nearly 100% (Figure 2A).

For ORC results, biotinylation was analyzed using the same assays. By comparing lanes 1, 2, and 3 in Figure 2B, it can be seen that biotinylated Orc1 was detected in lane 2 using the streptavidin-HRP (Figure 2B, top panel). Furthermore, a shift of biotinylated Orc1 in the presence of streptavidin was detected using an anti-CBP antibody. By comparing lanes 2 and 3 in Figure 2B (bottom panel), it can be seen that the band of Orc1 up-shifted by about 91%. These results indicate that approximately 91% of Orc1 was biotinylated *in vivo* (Figure 2B, bottom panel).

### Single-molecule observation of interaction between Qdot-labeled RPA and ssDNA

**Figure 3:**
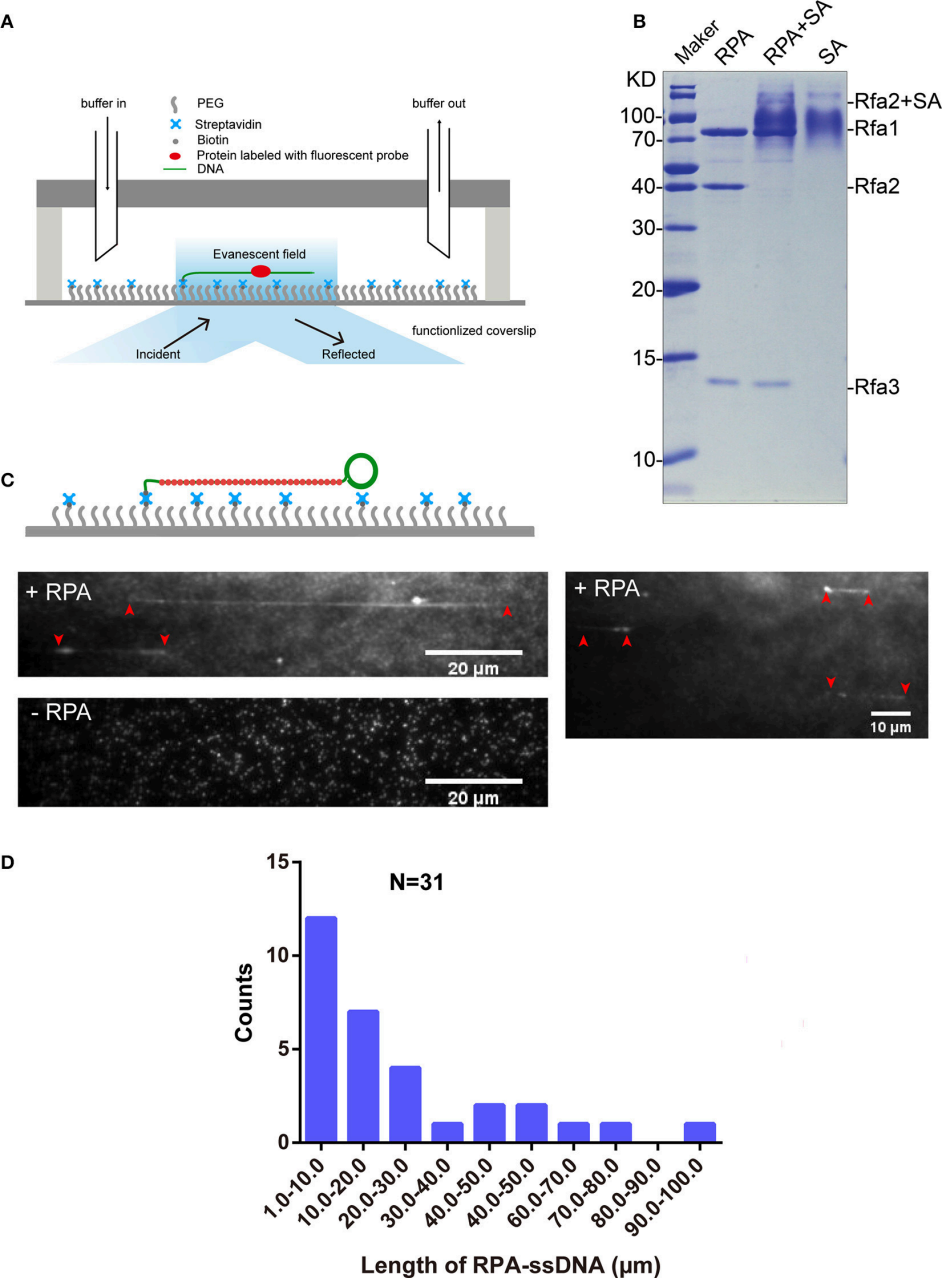
RPA purification and single-molecule visualization. **(A)** Single-molecule experiment platform. DNA was tethered on the coverslip through a biotin–streptavidin linkage. A protein bound on DNA is illustrated. Flow is from left to right. **(B)** Purified RPA and its biotinylation extent evaluation. Purified RPA (lane 1); RPA + unboiled streptavidin (SA) (lane 2); unboiled streptavidin (lane 3). Purified biotinylated-Rfa2 was shifted almost completely in the presence of streptavidin. **(C)** Illustration of streptavidin-coated-Qdot_705_ labeled RPA (RPA-Qdot_705_, red) binding on and stretching ssDNA (green), together with flow (top), 0.1 nM RPA-Qdot_705_ was pumped into flow cell (middle). 0.1 nM streptavidin-coated-Qdot_705_ was pumped into flow cell (bottom). Red arrows point out the two ends of RPA-ssDNA. **(D)** Length of ssDNA bound with RPA.

We used a microfluidic flow cell and TIRFM to observe DNA-protein interactions (Figure 3A). To observe the interaction between Qdot-labeled RPA and ssDNA at the single-molecule level, we first purified biotinylated RPA via tandem affinity purification with the aid of CBP tag and 3×Flag tag from the yeast homogenate. The extent of biotinylation of purified proteins was evaluated using the streptavidin shift assay. Figure 3B shows that Rfa1, 2, and 3 were successfully purified from budding yeast (Figure 3B, lane 1) and that biotinylated Rfa2 was totally shifted in the presence of excessive streptavidin (Figure 3B, lane 2). This shows that the biotinylation of purified RPA was also complete, which is consistent with the immunoblotting result shown in Figure 2A.

5′ biotin labeled ssDNA was attached to the coverslip via biotin–streptavidin linkage. RPA was labeled with Qdot_705_ by incubating biotinylated RPA with streptavidin-coated-Qdot_705_ in a 1:1 molar ratio for 10 min at room temperature. As expected, when 0.1 nM RPA-Qdot_705_ was pumped into the flow cell and incubated for 5 min, fluorescent signals of RPA-Qdot_705_ binding on ssDNA, which was stretched by 100 μl/min flow, were observed using a 405 nm laser and recorded by EM-CCD (Figure 3C, top panel, Movies S1, S2). To rule out the possibility that fluorescent signals were derived from nonspecific binding of streptavidin-coated-Qdot_705_ on ssDNA, 0.1 nM streptavidin-coated-Qdot_705_ was pumped into the flow cell. After 5 min incubation and flushing out of excessive Qdot_705_, no positive fluorescent signal on ssDNA was observed except for some background Qdot signals (Figure 3C, bottom panel, Movie S3). The background Qdots signals were due to direct interactions between streptavidin-coated Qdots and the biotinylated PEG on the coverslip. Based on the previous study's biochemistry results, RPA has an apparently higher affinity for single-stranded DNA than double-stranded DNA (Kim et al., 1992). We next used biotinylated native λDNA as substrates, and pumped 0.1 nM RPA-Qdot_705_ into the flow cell. After 5 min incubation, no Qdot_705_ fluorescent signals on λDNA were observed (Figure S3, Movies S4, S5). Therefore, streptavidin-coated-Qdot-labeled RPA can efficiently bind to ssDNA, and the binding of ssDNA-RPA-Qdot_705_ was successfully visualized by TIRFM. RPA binding on ssDNA can both remove secondary structures in ssDNA (San Filippo et al., 2008) and protect ssDNA. In our experiments, the lengths of ssDNA bound with RPA were distributed in the range of 1–100 μm (Figure 3D). Since ssDNA length can be stretched to 1.7 times the length of B-form DNA (Fu et al., 2011), the length of those ssDNAs in the flow cell were estimated to range from 1, 730 to 173, 000 nt. It is likely that fluorescent-probe labeled RPA will enable us to calculate the exact length of single-strand DNA in single-molecule experiments related to DNA replication and DNA damage repair.

### Biotinylated-ORC purification and single-molecule observation with Qdots

**Figure 4:**
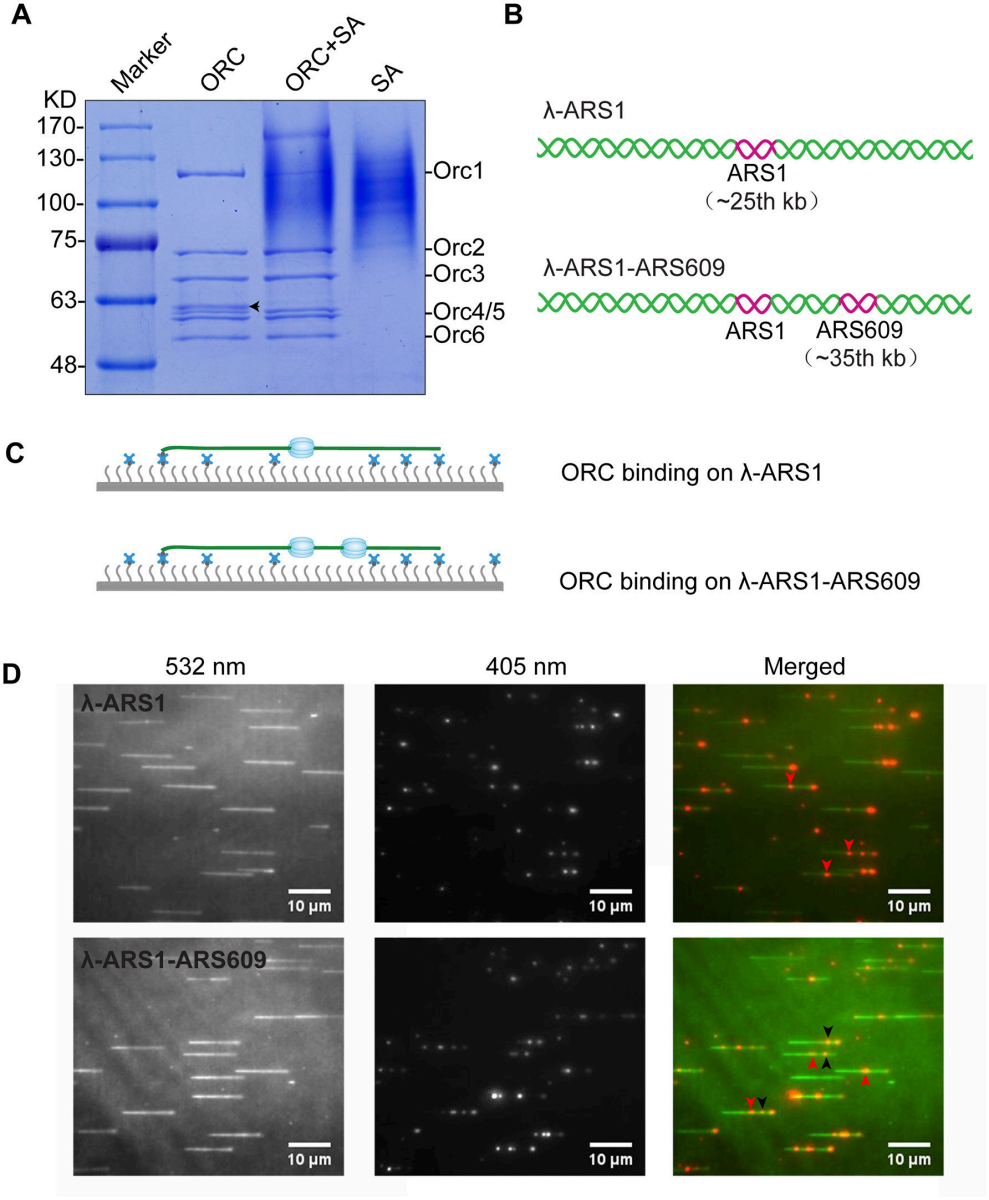
ORC purification and single-molecule visualization. **(A)** Purified ORC and its biotinylation efficiency evaluation. Purified ORC (lane 1); ORC + unboiled streptavidin (SA) (lane 2); unboiled streptavidin (lane 3). 90% Orc1 was shifted in the presence of streptavidin in lane 3, which was quantified using Image J. The band appearing between Orc3 and Orc4/5 in lane 1 of **(A)**, indicated by a black arrow, was identified as the degradation band of Orc1 using MALDI-TOF mass spectrometry. **(B)** DNA substrates for ORC binding. An 838 bp DNA fragment containing ARS1 sequence was inserted into native lambda DNA at *XbaI* site (24, 508 bp) and named λ-ARS1; a 715 bp DNA fragment containing ARS609 sequence was inserted into at *XhoI* site (34, 336 bp) of λ-ARS1 and named λ-ARS1-ARS609. **(C)** ORC-Qdot_705_ binding on λ-ARS1/λ-ARS1-ARS609. DNA tethered on coverslip with the aid of the biotinylated oligonucleotide complementary to the left end of native λ DNA, together with flow in theory. **(D)** ORC-Qdot_705_ binding on λ-ARS1 (top) and λ-ARS1-ARS609 (bottom). 0.5 nM ORC-Qdot_705_ was pumped into a flow cell with binding buffer. (Left) DNA was stained using SYTOX Orange and excited using a 532 nm laser; (center) ORC-Qdot_705_ was excited using a 405 nm laser; (right) merged images. Three ORC binding positions at ARS1 and ARS609 inserted sites are indicated by red and black arrows, respectively.

We next wanted to know whether our labeling system could detect the binding efficiency of ORC on different ARS. We therefore purified ORC by affinity purification using the CBP tag. The results of ORC purification are shown in lane 1 of Figure 4A. About 90% of Orc1 was up-shifted in the presence of streptavidin (Figure 4A, lane 2), which is consistent with the Western blotting results shown in Figure 2B.

**Figure 5:**
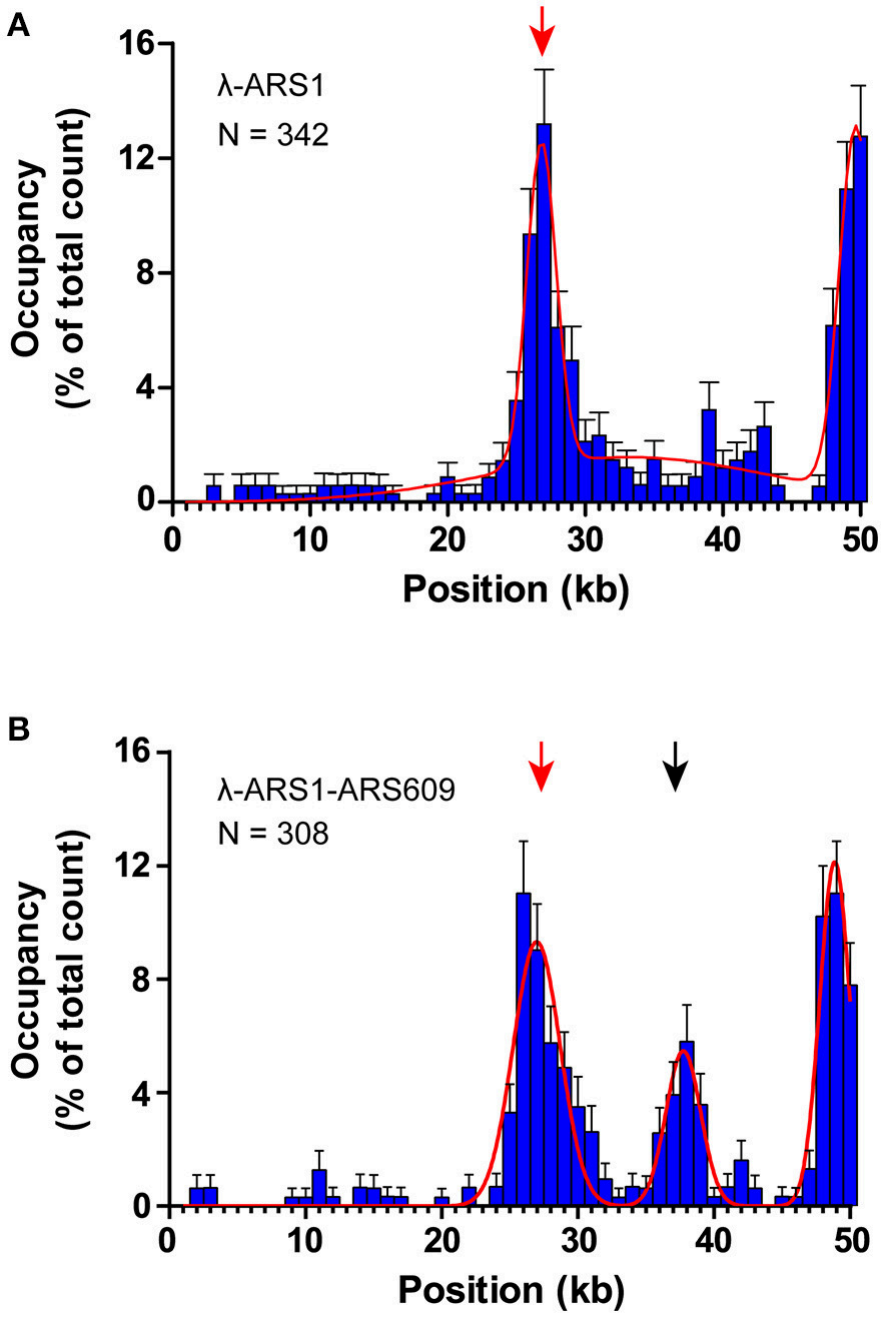
Distributions of ORC binding positions on λ-ARS1 and λ-ARS1-609. **(A)** Histogram and Gaussian fitting of ORC binding distributions on λ-ARS1. The position of the ARS1 inserted site is indicated by a red arrow. Data were analyzed using R and GraphPad Prism, and an error was defined based on 1000 bootstrap samples and a 95% confidential interval. *N* stands for the number of ORC molecules. **(B)** Histogram and Gaussian fitting of ORC binding distribution on λ-ARS1-ARS609. The positions of ARS1 and ARS609 inserted sites are indicated by red and black arrows, respectively.

To observe interactions between Qdot-labeled ORC and replication origins at the single-molecule level, λ-ARS1 and λ-ARS1-ARS609, which were acquired by inserting ARS1 or both ARS1 and ARS609 into native λDNA, were utilized as DNA substrates (Figure 4B). λ-ARS1 and λ-ARS1-ARS609 were biotinylated by annealing with abiotinylated oligonucleotide (5′-AGGTCGCCGCC-TEG-Biotin-3′) complementary to the left end of native λDNA (Yardimci et al., 2012), and singly tethered on the coverslip through a biotin–streptavidin linkage with the blocking buffer. Next, ORC-biotin was labeled with streptavidin-coated Qdot_705_ at a 1:1 molar ratio for 10 min at room temperature, and 0.5 nM Qdot_705_ labeling ORC was pumped into the flow cell with binding buffer. After excessive proteins were flushed out by the binding buffer, fluorescent signals of ORC-Qdot_705_ were observed using a 405 nm laser at 100 μl/min flow. DNA was stained with SYTOX Orange and observed with a 532 nm laser. Interactions of ORC and λ-ARS1/λ-ARS1-ARS609 were concurrently observed using W-View Gemini Imaging splitting optics and images were recorded by EM-CCD. Differently from the expected binding distribution (Figure 4C), ORC-Qdot_705_ proteins not only bound on the inserted ARS sites but also bound on some other sites on λDNA (Figure 4D, Movies S6–S11). After careful sequences analysis, we found that the other ORC-Qdot_705_ binding sites were all AT-rich (Figure S4), which is an important characteristic of ARS sequences (Bell and Labib, 2016). To determine the distribution of ORC on λ-ARS1/λ-ARS1-ARS609, the ORC binding positions on the intact singly tethered DNA substrates were quantitatively analyzed. For the λ-ARS1 substrate, 342 binding positions of ORC-Qdot_705_ molecules on 187 λ-ARS1 molecules were analyzed (Data Sheet 1 in Supplementary Material). It showed that ORC-Qdot_705_ binds with high abundance at both the ARS1 site (approximately at 27 kb) and the free end of DNA (approximately at 50 kb) (Figure 5A). ORC-Qdot_705_ binding positions (12.8%) were distributed at the ARS1 site, and 13% of the positions were at the free end of the λ-ARS1 DNA. For the λ-ARS1-ARS609 substrate, 308 binding positions of ORC-Qdot_705_ molecules on 138 λ-ARS1-ARS609 molecules were analyzed (Data Sheet 2 in Supplementary Material). ORC-Qdot_705_ also binds to the ARS1 site and the free end of DNA with high efficiency. ORC-Qdot_705_ binding positions (9.3%) were at the ARS1 site (approximately at 27 kb), and 12.8% positions were at the free end (approximately at 49 kb). For the ARS609 sites, the ORC binding occupancy was 5.4%, which is less than the occupancy at the ARS1 site. Comparing the distributions on λ-ARS1 and λ-ARS1-ARS609, we can observe the obvious specificity for ORC-Qdot_705_ binding at ARS609 (approximately at 38 kb) (Figure 5B). Regarding the different binding abundances of ORC-Qdot_705_ in different ARS; it could be that different ARS have different capabilities to recruit the ORC complex. It is likely that early firing origins (for example, ARS1) recruit the ORC more efficiently than late firing origins (for example, ARS609). However, owing to the resolution limitations of microscopy, we cannot rule out the possibility that the binding abundance at the ARS1 site resulted from the addition effects of both the ARS1 site and other AT-rich sites that are close to the ARS1 inserted site. This could have occurred since there is a high abundance of ORC binding on the AT-rich sites located in the central part of native λ DNA (Figure S4; Duzdevich et al., 2015).

**Figure 6:**
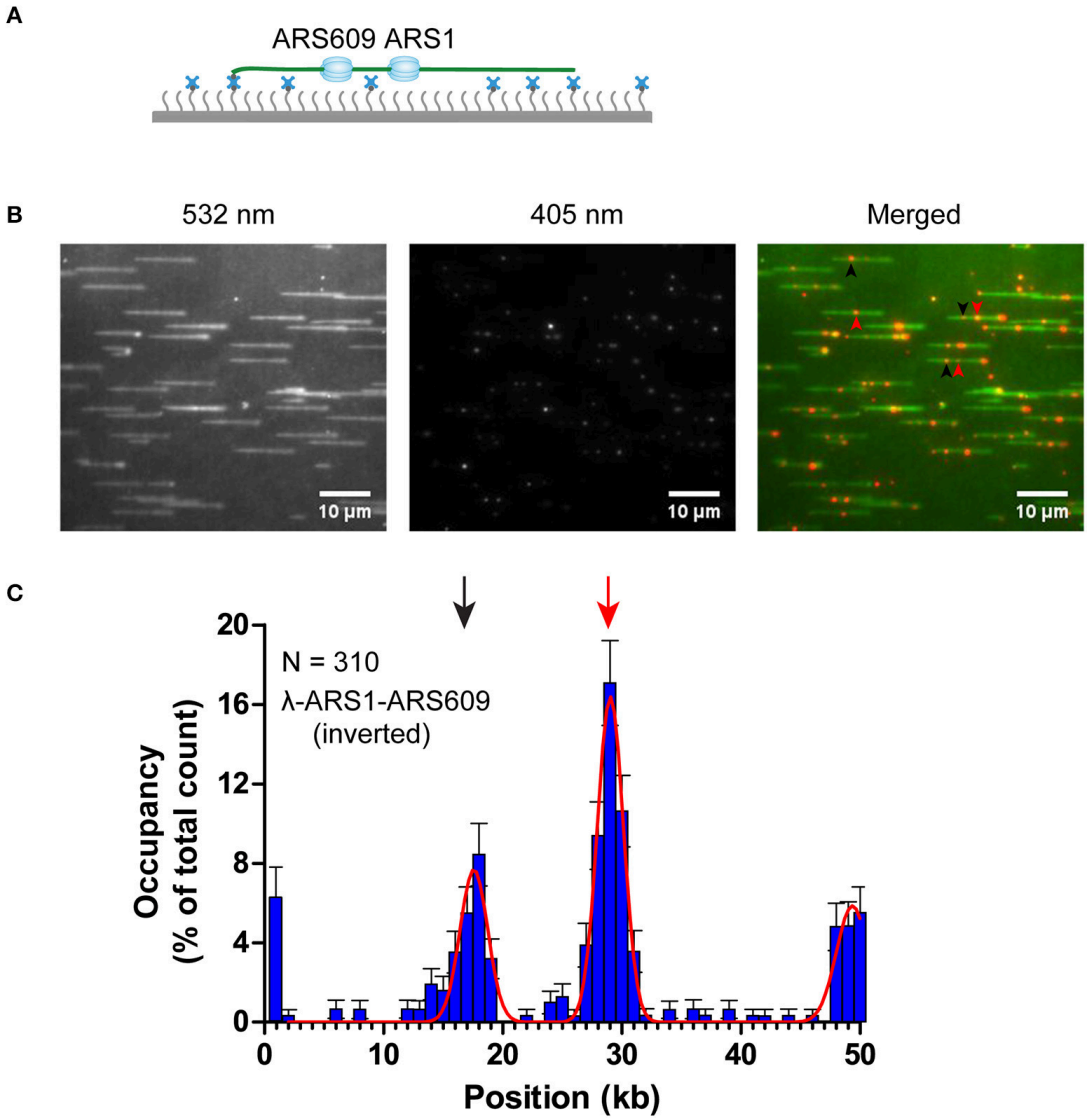
ORC binding on inverted λ-ARS1-ARS609. **(A)** ORC binding on inverted λ-ARS1-ARS609 together with flow in theory. DNA was biotinylated using the biotinylated oligonucleotide complementary to the right end of native λDNA. **(B)** ORC-Qdot_705_ binding on inverted λ-ARS1-ARS609. (left) DNA was stained using SYTOX Orange and excited using a 532 nm laser; (center) ORC-Qdot_705_ was excited using a 405 nm laser; (right) merged images. Three ORC binding positions at ARS1 and ARS609 are indicated by red and black arrows, respectively. **(C)** Distribution of ORC binding on inverted λ-ARS1-ARS609. Positions of ARS1 and ARS609 inserted sites are indicated by red and black arrows, respectively.

The theoretical contour length of B-form λ-ARS1-ARS609 is 17 μm, but the singly tethered λ-ARS1-ARS609 extended only to 13.2 ± 0.33 μm at 100 μl/min flow rate, which was around 80% of the predicted length (Figure S5). Thus, we noticed that the positions of ORC binding at the ARS1 and ARS609 sites that we had observed in our experiments had shifted 2–4 kb to the right side compared with the theoretical sites (Figure 5B). Moreover, the free end of the DNA molecule was stretched less than the tethered end because the fluidic force act less efficiently on the free end in the flow cell. This may explain why ORC obviously prefers to bind the free DNA end that is located after the ARS sites. The strong ORC signal at the free end of the DNA might have resulted from the accumulation of ORC binding at all the AT-rich regions near the less-stretched free end. To obtain further evidence for our explanation, we biotinylated λ-ARS1-ARS609 using a biotinylated oligonucleotide (5'-GGGCGGCGACCT-TEG-Biotin-3') complementary to the right end of native λDNA and then observed the interaction between the ORC and DNA at the single-molecule level (Figure 6A). 310 binding positions of ORC-Qdot_705_ molecules on 165 λ-ARS1-ARS609 (inverted) molecules were analyzed (Data Sheet 3 in Supplementary Material). As expected, the strong ORC signal switched from the right of the ARS1 to the left (Figures 6B,C, Movies S11 to S14), which was the new location of ARS609 (approximately at 18 kb). Meanwhile, the frequency of ORC binding at the free end of DNA (approximately at 49 kb) decreased from 12.8% (Figure 5B) to 5.8% (Figure 6C), which is consistent with the fact that there are more AT-rich sites closer to the right end of λDNA than there are closer to the left end (Figure S4; Duzdevich et al., 2015).

## Discussion

In this study, we established a simple system to biotinylate a target protein at a specific site in budding yeast, purify the protein after the BirA–Avi based biotinylation *in vivo*, and label it with Qdots conveniently through the biotin–streptavidin interaction. As a proof-of-concept, two protein complexes (RPA and ORC) were purified, and their interactions with DNA were successfully observed by TIRFM at the single-molecule level.

We constructed the strains integrated with *GAL1-BIRA* or *GAL1-NLS-BIRA*, and the expressing plasmids with Avi-tag at the N- or C- termini. The strain bearing *GAL1-BIRA* was used for biotinylating the proteins located in cytoplasm, such as RPA, and *GAL1-NLS-BIRA* was used for biotinylating proteins located in nuclei, such as ORC. CBP or 3×Flag tag was used in tandem with Avi-tag for protein affinity purification; this avoids contamination of proteins expressed from genes at native genomic loci. Accordingly, a high extent of biotinylation of target proteins was achieved. Biotin, as the substrate of biotinylation process, needs to be supplemented in SC-medium (van Werven and Timmers, 2006). In this study, yeast cells were cultured in YP-medium. We found that the concentration of biotin in YP-medium was sufficient to support overexpressed protein biotinylation.

Compared with Avi-tag biotinylated by BirA enzyme *in vitro* (Kad et al., 2010), BirA co-expressed with Avi-tagged target proteins *in vivo* is economic and convenient as it does not need to be purified, does not require a change of buffers in order to be suitable for biotinylation reaction, and does not require the protein to be purified again to remove excess biotin. As reported in other studies (Forejtnikova et al., 2010; Nie and Kaback, 2010), biotinylated Avi has also been used as a tag to purify proteins using monomeric avidin agarose in mammalian cells or *E. coli*. However, this purification method should be used with caution in biotinylated proteins purification and streptavidin-coated Qdots labeling assays. First, except for the recombinant biotinylated protein, there are some endogenous biotinylated proteins in yeast (Kim et al., 2004; van Werven and Timmers, 2006). Second, excess biotin from the step of protein elution must be removed thoroughly before carrying out subsequent assays such as biotinylation quantitation and streptavidin-coated-Qdot labeling.

Fluorescent proteins are a good tool for tagging target proteins in yeast, owing to the development of powerful genetic manipulation techniques in the past (Boeke et al., 1984, 1987; Longtine et al., 1998; Wang et al., 2017). However, compared with organic dyes and Qdots, both the lower intensity and stability of fluorescent proteins restricts them for use in single-molecule studies. The Qdot is an ideal fluorescent probe for studying DNA–protein interactions at the single-molecule level, owing to its high brightness and photo-bleaching resistance. Two common methods have been reported to label target proteins with Qdots. One involves labeling with antibody-coated Qdots (Sternberg et al., 2014); the other involves labeling with streptavidin-coated Qdots as described in this study. Compared with antibody-coated Qdots, labeling with streptavidin-coated Qdots has distinct advantages: (1) this method can be used more universally for different protein labeling than the primary antibody-coated Qdots; (2) the presently used method for labeling antibodies with Qdots is complex, expensive, and time-consuming. Moreover, the antibody labeling efficiency is normally not high enough for a common biochemistry laboratory; and (3) the labeling antibodies must be stored at 4°C because Qdots cannot be frozen, but many antibodies should be stored at −20°C to ensure conservation for long periods. During the labeling of the protein with streptavidin-coated Qdots, it is possible that some fraction of Qdots are bound with more than one protein since there are approximately 5–10 streptavidins per Qdot nanocrystal according to the manufacture's manual (Thermo Fisher Scientific). Thus, when carrying out labeling assays, the appropriate molar ratio of proteins and streptavidin-coated Qdot should be carefully chosen. Moreover, extreme caution should be taken when the exact number of labeled protein molecule needs to be calculated. Another key point that should be emphasized in this method described is that both the protein labeling and the DNA tethering are based on the interaction between biotin and streptavidin; thus, the biotinylated PEG should be blocked sufficiently using streptavidin before proteins labeled with streptavidin-coated Qdots are pumped into the flow cell.

Organic dyes, as mentioned, are a class of commonly used fluorescent probe. Frequently used site-specific organic-dye labeling methods include labeling Cy3 or Cy5 at a cysteine residue (Lin and Wang, 2008) and labeling Alexa derivatives using SNAP, Halo tag, or Sort tag (Stratmann and van Oijen, 2014; Duzdevich et al., 2015; Ticau et al., 2015, 2017). In this study, our system can also be utilized to tag biotinylated target proteins with organic dyes using commercially available organic-dye labeled streptavidin. Moreover, in addition to studying DNA–protein interactions by tethering single- or double-strand DNA on a functionalized coverslip (Redding et al., 2015; Ticau et al., 2015; Yeeles et al., 2015; Qi and Greene, 2016), it is also an elegant strategy to study protein–protein or RNA–protein interactions by tethering biotinylated proteins on a coverslip through a biotin–streptavidin linkage (Lu et al., 2015a,b; Fareh et al., 2016). Therefore, we are confident that with the rapid development and wide application of single-molecule microscopy, the method described in this paper will be of great benefit to future research in this field.

## Author contributions

HX, YB, and YF designed the experiments. HX, YB, ZZ, XC, and XX performed the experiments. HX, YB, ZZ, XC, and YF analyzed the data. HX, YB, and YF prepared the manuscript.

### Conflict of interest statement

The authors declare that the research was conducted in the absence of any commercial or financial relationships that could be construed as a potential conflict of interest.
