# Supplementary material for: Utilizing Biotinylated Proteins Expressed in Yeast to Visualize DNA–Protein Interactions at the Single-Molecule Level
Source: Front Microbiol. 2017 Oct 24;8:2062. doi: 10.3389/fmicb.2017.02062 (PMC5662892; doi:10.3389/fmicb.2017.02062)
Supplement: Supplementary file 1 [file Image1.PDF]

Supplementary Material

Utilizing Biotinylated Proteins Expressed in Yeast to Visualize DNA–Protein Interactions at the Single-Molecule Level

Huijun Xue[1,2], Yuanyuan Bei[1,2], Zhengyan Zhan[1], Xiuqiang Chen[1,2], Xin Xu[1], Yu V. Fu[1,2*]

*** Correspondence:** Yu V. Fu: fuyu@im.ac.cn

A

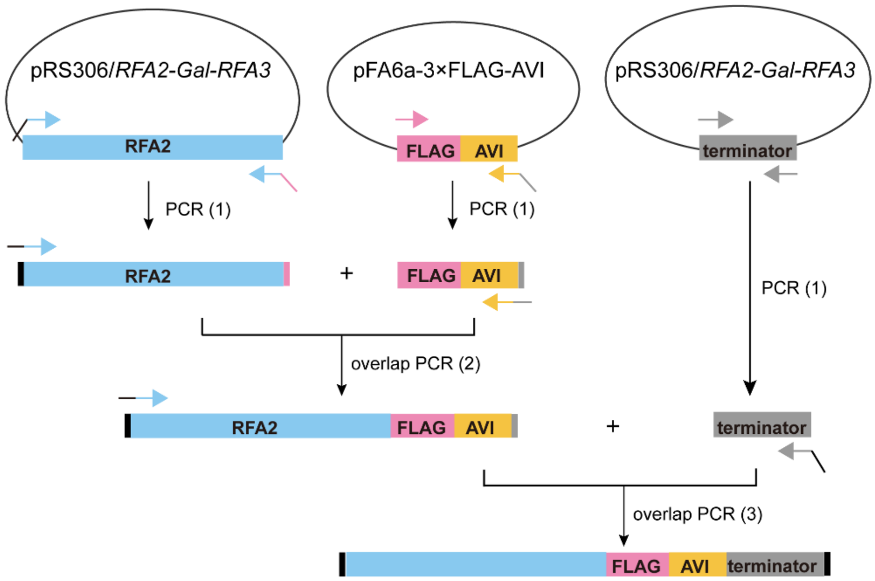

B

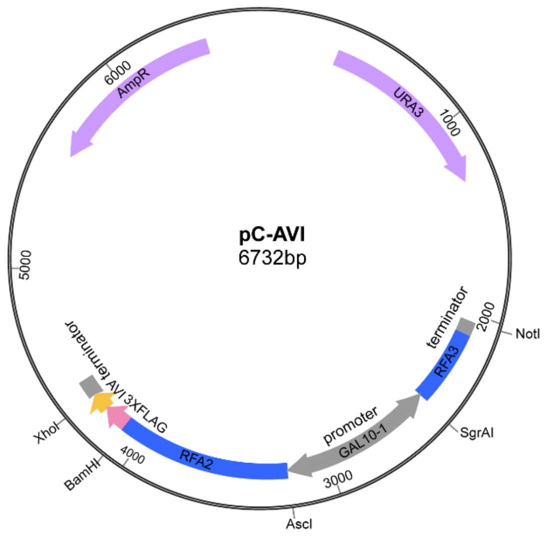

Supplementary Figure 1. Plasmid construction of pC-AVI. **Related to Figure 1**.
(A) AVI tag was added at C terminus of *RFA2* by overlap PCR. 1.) *3×FLAG-AVI* fragment was amplified from pFA6a-3×FLAG-AVI, and *RFA2* gene and a terminator were amplified from pRS306/*RFA2-Gal-RFA3*, respectively; 2.) *RFA2-3×FLAG-AVI* was amplified by overlap PCR; 3.) *RFA2-3×FLAG-AVI*-terminator was amplified by overlap PCR. 20 bp overlapping sequences at the end of DNA fragments were showed in pink and gray. 15-20 bp homologous sequences of the two ends of *AscI-XhoI* digested pRS306/*RFA2-Gal-RFA3*, were added at the ends of *RFA2-3×FLAG-AVI*-terminator by PCR and showed in black. **(B)** Plasmid map of pC-AVI.
