# Supplementary material for: Utilizing Biotinylated Proteins Expressed in Yeast to Visualize DNA–Protein Interactions at the Single-Molecule Level
Source: Front Microbiol. 2017 Oct 24;8:2062. doi: 10.3389/fmicb.2017.02062 (PMC5662892; doi:10.3389/fmicb.2017.02062)
Supplement: Supplementary file 2 [file Image2.PDF]

Supplementary Material

Utilizing Biotinylated Proteins Expressed in Yeast to Visualize DNA–Protein Interactions at the Single-Molecule Level

Huijun Xue[1,2], Yuanyuan Bei[1,2], Zhengyan Zhan[1], Xiuqiang Chen[1,2], Xin Xu[1], Yu V. Fu[1,2*]

*** Correspondence:** Yu V. Fu: fuyu@im.ac.cn

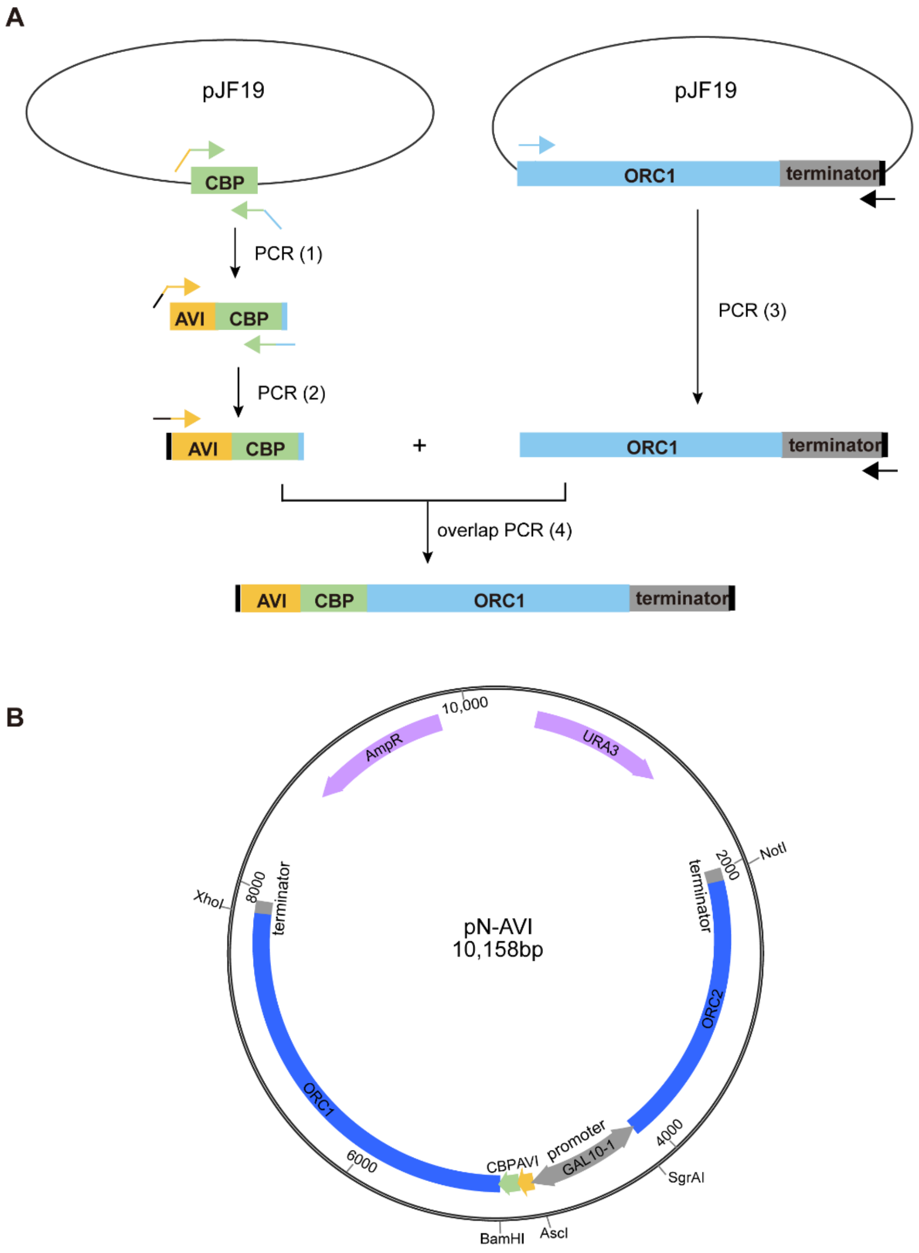

Supplementary Figure 2. Plasmid construction of pN-AVI. Related to Figure 1.
(A) AVI tag was added at N terminus of *ORC1* by overlap PCR. 1-2.) *AVI-CBP* was amplified from pJF19 by overlap PCR; 3.) *CBP-ORC1*-terminator was amplified from pJF19; 4.) *AVI-CBP-ORC1*-terminator was amplified by overlap PCR. A 20 bp overlapping sequence at the end of *AVI-CBP* was showed in blue. 15-20 bp homologous sequence of the two ends of *AscI-XhoI* digested pJF19, were added at at the ends of *AVI-CBP-ORC1*-terminator and showed in black. **(B)** Plasmid map of pN-AVI.
