# Supplementary material for: Utilizing Biotinylated Proteins Expressed in Yeast to Visualize DNA–Protein Interactions at the Single-Molecule Level
Source: Front Microbiol. 2017 Oct 24;8:2062. doi: 10.3389/fmicb.2017.02062 (PMC5662892; doi:10.3389/fmicb.2017.02062)
Supplement: Supplementary file 3 [file Image3.PDF]

Supplementary Material

Utilizing Biotinylated Proteins Expressed in Yeast to Visualize DNA–Protein Interactions at the Single-Molecule Level

Huijun Xue[1,2], Yuanyuan Bei[1,2], Zhengyan Zhan[1], Xiuqiang Chen[1,2], Xin Xu[1], Yu V. Fu[1,2*]

*** Correspondence:** Yu V. Fu: fuyu@im.ac.cn

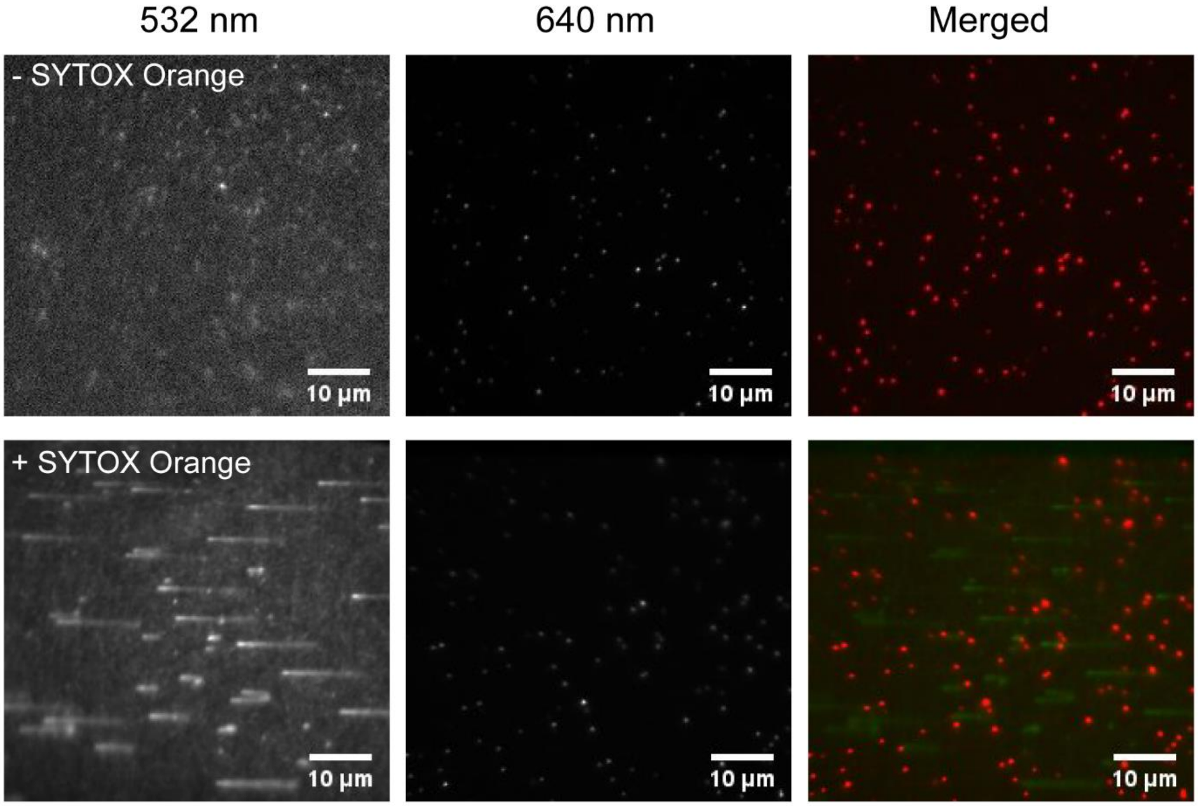

Supplementary Figure 3. 0.1 nM RPA-Qdot$_{705}$ can not bind on native λDNA. **Related to Figure 3**.

RPA-Qdot$_{705}$ did not bind on dsDNA. Native λDNA molecules were tethered on the coverslip firstly, then 0.1 nM RPA-Qdot$_{705}$ was pumped into a flow cell. Images were recorded before (top panel) and after (lower panel) staining DNA using SYTOX orange. (left) λDNA was excited using a 532 nm laser; (center) Qdot$_{705}$ was excited using a 405 nm laser; (right) merged images. The Qdot$_{705}$ signals were background, because they did not move along with λDNA stretched by 100 µl/min flow, which can be seen in Movie S4-5.
