# Supplementary material for: Utilizing Biotinylated Proteins Expressed in Yeast to Visualize DNA–Protein Interactions at the Single-Molecule Level
Source: Front Microbiol. 2017 Oct 24;8:2062. doi: 10.3389/fmicb.2017.02062 (PMC5662892; doi:10.3389/fmicb.2017.02062)
Supplement: Supplementary file 4 [file Image4.PDF]

Supplementary Material

Utilizing Biotinylated Proteins Expressed in Yeast to Visualize DNA–Protein Interactions at the Single-Molecule Level

Huijun Xue[1,2], Yuanyuan Bei[1,2], Zhengyan Zhan[1], Xiuqiang Chen[1,2], Xin Xu[1], Yu V. Fu[1,2*]

*** Correspondence:** Yu V. Fu: fuyu@im.ac.cn

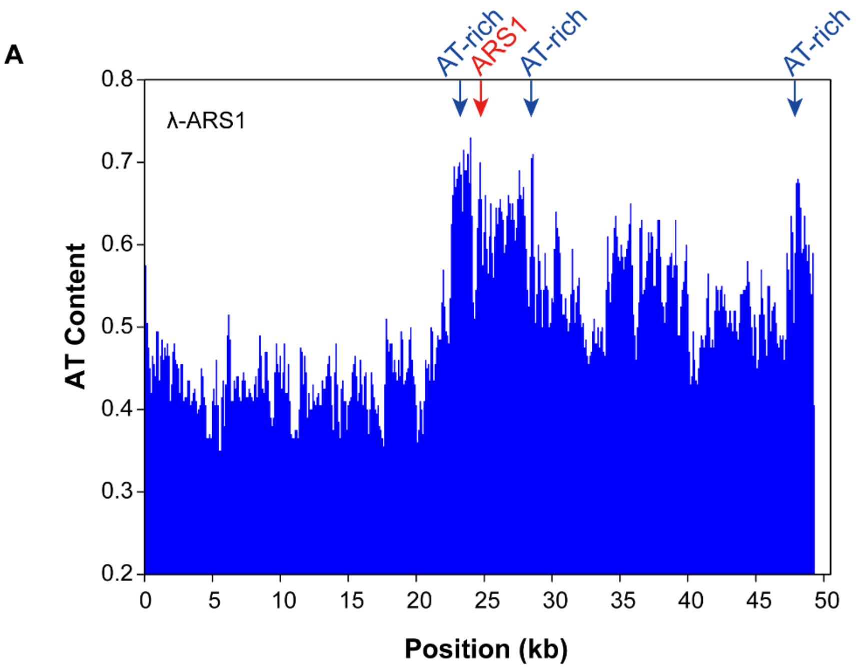

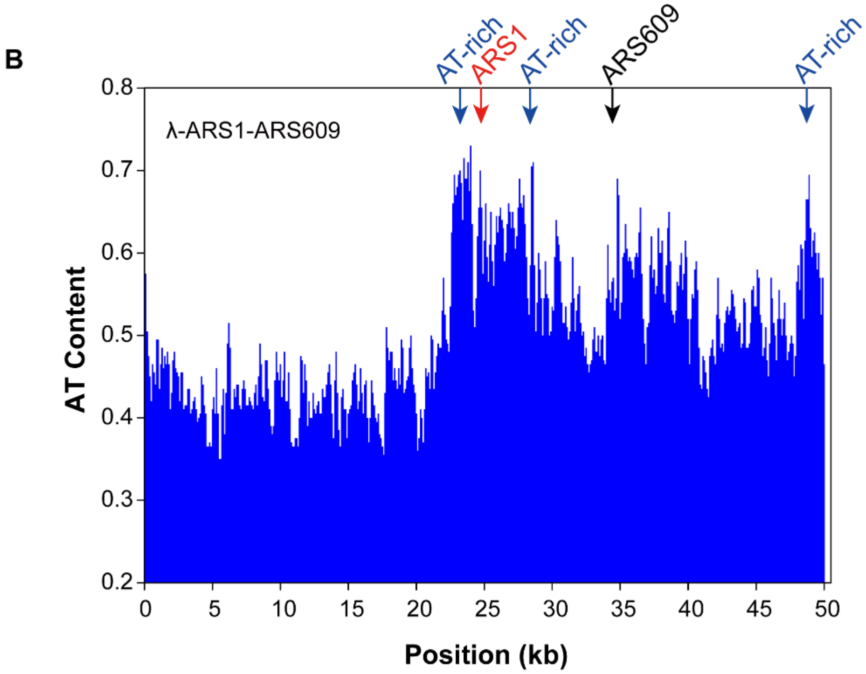

Supplementary Figure 4. AT content of λ-ARS1 and λ-ARS1-ARS609. **Related to Figure 5 and 6**.

AT content of **(A)** λ-ARS1 and **(B)** λ-ARS1-ARS609 was analyzed by binned to 200bp. The sites of cloned ARS1, ARS609 and AT-rich were pointed out using red, black and blue arrows, respectively.
