# Supplementary material for: Utilizing Biotinylated Proteins Expressed in Yeast to Visualize DNA–Protein Interactions at the Single-Molecule Level
Source: Front Microbiol. 2017 Oct 24;8:2062. doi: 10.3389/fmicb.2017.02062 (PMC5662892; doi:10.3389/fmicb.2017.02062)
Supplement: Supplementary file 5 [file Image5.PDF]

Supplementary Material

Utilizing Biotinylated Proteins Expressed in Yeast to Visualize DNA–Protein Interactions at the Single-Molecule Level

Huijun Xue[1,2], Yuanyuan Bei[1,2], Zhengyan Zhan[1], Xiuqiang Chen[1,2], Xin Xu[1], Yu V. Fu[1,2]*

*** Correspondence:** Yu V. Fu: fuyu@im.ac.cn

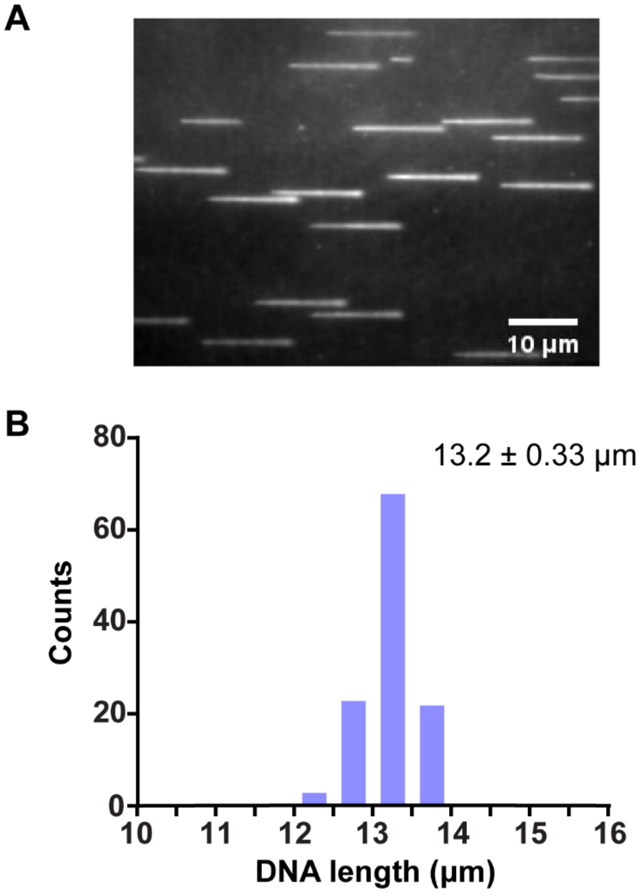

Supplementary Figure 5. Length of DNA. **Related to Figures 4 to 6**.
(A) Singly tethered λ-ARS1-ARS609. DNA was tethered at the left end, stretched with 100 µl/min flow, and stained using SYTOX Orange. 61 sequential images from its corresponding stack were processed by average intensity type of Z projection. **(B)** Histogram of DNA length; DNA length was 13.2 ± 0.33 µm at 100 µl/min flow rate.
