# Supplementary material for: Utilizing Biotinylated Proteins Expressed in Yeast to Visualize DNA–Protein Interactions at the Single-Molecule Level
Source: Front Microbiol. 2017 Oct 24;8:2062. doi: 10.3389/fmicb.2017.02062 (PMC5662892; doi:10.3389/fmicb.2017.02062)
Supplement: Supplementary file 6 [file Table1.DOCX]

**Table S1.** Primers for pN-AVI and pC-AVI construction^a^.

| **Plasmid construction Primers** | **Sequences (5’→3’)** |
| --- | --- |
| **pN-AVI** |  |
| RFA2-F | **TTGAAAATTCAATATAA**GGCGCGCCTATAAAACAATGGCTACTTACC |
| RFA2-R | TCATGGTCTTTGTAGTCCATGGATCCCAAAGCGAAGAAGTTGTTATCATCG |
| FLAG-AVI-F | GACTACAAAGACCATGACGG |
| FLAG-AVI-R | AAAAAATTGATCTATCGATTTCATTCGTGCCATTCGATTT |
| Terminator-F | AATCGATAGATCAATTTTTTTCTTTTCTC |
| Terminator-R | GCATAAAGGCATTAAAAGAGGAG |
| Final-F  (same as RFA2-F) | **TTGAAAATTCAATATAA**GGCGCGCCTATAAAACAATGGCTACTTACC |
| Final-R | **GGTACCGGGCCCCCC**CTCGAGGCATAAAGGCATTAAAAGAGGAG |
| **pC-AVI** |  |
| AVI-CBP-F1 | CATCTTCGAGGCTCAGAAAATCGAATGGCACGAATATAAAACAATGAAGAGAAGATGG |
| AVI-CBP-F2 | GGCGCGCCATGTCCGGCCTGAACGACATCTTCGAGGCTCAGAAAATC |
| AVI-CBP-R | TCCTTCAAAGTCTTAGCCATGGATCCCAAAGCACCAGAAGAAGAAATCTTC |
| ORC1-terminator-F | ATGGCTAAGACTTTGAAGGACTTGCAAGGTTGGGAAATTATTAC |
| ORC1-terminator-R | GGTACCGGGCCCCCCCTCGAG |
| Final-F | **TTGAAAATTCAATATAA**GGCGCGCCATGTCCGGCC |
| Final-R  (same as  ORC1-terminator-R) | **GGTACCGGGCCCCCC**CTCGAG |

^a^ Overlap sequences were indicated using underline. Homologous sequences of vectors were indicated in bold.
